# Redox mechanisms of conversion of Cr(VI) to Cr(III) by graphene oxide-polymer composite

**DOI:** 10.1038/s41598-020-65534-8

**Published:** 2020-06-08

**Authors:** P. C. Bandara, J. Peña-Bahamonde, D. F. Rodrigues

**Affiliations:** Department of Civil and Environmental Engineering, University of Houston, Houston, TX 77204-4003 USA

**Keywords:** Chemistry, Materials science, Nanoscience and technology

## Abstract

Alternative methods of aqueous chromium removal have been of great research interest in recent years as Cr (VI) is a highly toxic compound causing severe human health effects. To achieve better removal of Cr (VI), it is essential to understand the chemical reactions that lead to the successful removal of Cr species from the solution. Recent studies have demonstrated that graphene oxide (GO) based polymer beads cannot only adsorb Cr (VI) via electrostatic attractions but also reduce it to Cr (III), which is a much less toxic form of chromium. This conversion and the functional groups involved in this conversion, until now, were not elucidated. In the present study, we employed X-ray photoelectron spectroscopy and Fourier-transform infrared spectroscopy to investigate the conversion pathway of Cr (VI) to Cr (III) in graphene-based polymer beads. The results showed that alcoholic groups are converted to carboxylic groups while reducing Cr (VI) to Cr (III). The inclusion of GO in the polymer beads dramatically increased the potential of Cr (VI) uptake and conversion to Cr (III), indicating polymers and nanomaterials containing alcohol groups can remove and convert chromium in water. Other functional groups present in the polymer bead play an important role in adsorption but are not involved in the conversion of Cr (VI) to Cr (III).

## Introduction

Chromium removal in water treatment is a big challenge since the maximum limit concentration allowed in drinking water is only 0.1 ppm. Cr can be found in many oxidation forms; Cr (VI) being the most toxic and soluble, and Cr (III), the least toxic form of chromium^1^. Conventionally, heavy metals are removed by techniques that produce hazardous chemical wastes and require post-treatment^2^. Therefore, a wider interest has been shown in finding alternative methods to remove Cr species from water to ensure sustainable and consumable water supply. In this premise, earlier studies have shown that various combinations of graphene oxide-based nanomaterials can effectively remove Cr (VI) ions. Furthermore, in many cases, Cr (VI) has shown to be reduced to Cr (III); however, the chemical pathway behind this removal and reduction has not been fully elucidated. Primarily, it was theorized to be a combination of electrostatic attraction and chemical complexation based on previous reports with other carbonaceous materials^3–6^. For instance, Chen *et al*. reported that Cr (VI), in the form of Cr_2_O_7_^2−^ anions bound to positively charged surfaces of wood-based powdered activated carbon (WPAC), was reduced to Cr (III), suggesting that some graphitic carbons in WPAC allowed a redox reaction to happen^5^. Similarly, several studies have indicated that the Cr (VI) ions in a solution can form ion complexes with graphene oxide functional groups or can be subjected to electrostatic attractions allowing easy removal from a solution by a CS-PEI-GO nanocomposite^3,4,6^. These studies showed the successful removal of Cr (VI) by carbon-based nanomaterials and polymer beads. Some of them reported the conversion of Cr (VI) to Cr (III), but none of them investigated the actual pathways of conversion of Cr (VI) to Cr (III) during the removal of Cr (VI).

Based on this knowledge gap, in the present study, we synthesized graphene oxide (GO) embedded in chitosan (CS) and polyethyleneimine (PEI) with the aid of a crosslinking reagent, glutaraldehyde (GLA)^3^. This material was selected since it was shown to remove Cr (VI) and convert to Cr (III) and because these beads contain diverse functional groups, such as amine, hydroxyl, carboxyl, carbonyl and alkyl groups, which were previously suggested to be playing a significant role in this conversion of Cr (VI) to Cr(III). The polymer beads were synthesized according to a previously reported method^3,7,8^.

## Materials

The GO used in the study was synthesized using the modified Hummer’s method^3,9^ starting from graphite (<45 µm) purchased from Sigma Aldrich. Polymeric materials, CS (low molecular weight), PEI (50% (wt/wt%) in water, avg. MW 750,000), and crosslinking agent GLA (25% (w/w%) in water) were purchased from Sigma Aldrich. To prepare the solutions containing Cr (VI), we used potassium dichromate (K_2_Cr_2_O_7_, crystals, 99.8% assay) purchased from Fisher Scientific (Hanover Park, IL, USA). The Cr (VI) solutions were prepared in deionized (DI) water filtered with a 0.2 µm polyethersulfone filter. The other reagents required for the bead synthesis, sodium hydroxide (NaOH, ACS reagent, ≥ 97% assay, pellets) and hydrochloric acid (HCl, ACS reagent, 37% assay), were purchased from Sigma Aldrich.

### CS-PEI-GO bead preparation

CS-PEI-GO polymer bead was synthesized according to the optimum composition suggested by Perez *et al.*^3^. Briefly, a solution of CS-PEI-GO containing 2.0% CS, 2.0% PEI and 1500 ppm GO were stirred overnight at 150 rpm to form a homogeneous solution. After that, the mixture was added to a 10 mL syringe (Becton, Dickinson and Company, Franklin Lakes, NJ) with a 23 G BD Precision Glide needle (Becton, Dickinson, and Company, Franklin Lakes, NJ) bent at 90° angle and was connected to a NE-300 Just Infusion syringe pump (New Era Pump Systems. Farmingdale, NY). The mixture in the syringe was carefully dropped at 1 mL/min feed rate to a precipitation bath containing 1.5 M NaOH to form spherical beads. The NaOH bath was stirred at 150 rpm to avoid aggregation of freshly formed beads. The beads were allowed to precipitate in the NaOH bath and collected after about 30 minutes. The beads were washed several times with DI water until the pH became neutral. Then, the CS-PEI-GO beads were crosslinked with 2.08% glutaraldehyde (GLA) for 30 minutes. Finally, the beads were washed with DI water to remove excess GLA. Negative control beads, containing CS and CS-PEI, were also prepared in a similar procedure as the CS-PEI-GO. For the control beads, CS and PEI concentrations were the same as the concentrations used for the CS-PEI-GO, but without adding GO or PEI.

### Characterization of GO and CS-PEI-GO beads

GO used in this study was synthesized using the modified Hummer’s method, and successful synthesis of GO was confirmed through characterization techniques: Attenuated total reflection Fourier-transform infrared spectroscopy (ATR-FTIR), X-ray photoelectron spectroscopy (XPS), scanning electron microscopy (SEM). Further information about GO and polymer beads synthesis and detailed characterizations are available in our previously published work^3,7,8^. In the present study, the synthesized CS-PEI-GO beads were characterized in detail with XPS and ATR-FTIR in order to identify the successful synthesis and functional variability of the beads before and after adsorption to Cr(VI) that could lead to the Cr species uptake and reduction. Prior to the XPS and ATR-FTIR analysis, the beads were freeze-dried for 12 h and ground to obtain a fine powder. For the XPS analysis, a PHI 5700 X-ray photoelectron spectrometer was used. Low resolution with pass energy of 23.5 eV was performed for the wide scan. Pass energy of 187.8 eV was used for high-resolution scans to acquire the photoelectron emissions from the used beads. Spectrum data were processed using the MultiPak V7.0.1 (ULVAC-PHI, Inc.) and Origin Pro8.5 (OriginLab, Northampton, MA). ATR-FTIR spectra of the ground polymer beads were analyzed with a Nicolet iS10 Mid Infrared FTIR Spectrometer (Thermo Fisher Scientific, USA) having air as the background. Acquired raw spectra were post-processed employing Omnic 8 Software (Thermo Fisher Scientific, USA) and Origin Pro8.5 software. Zeta-potential measurements were performed in a Zetasizer Nano (Malvern) using the zeta potential transfer standard DTS 1235. The samples were measured with 100 mg/L of each sample in DI water at pH 3.

### Adsorption of Cr and XPS analysis

Amounts of 0.5 g of wet CS, CS-PEI, and CS-PEI-GO were measured and used for the batch treatment with 50 mL of 100 ppm Cr (VI) solution (pH~3). The natural pH of the Cr (VI) solutions was used; therefore, no pH adjustment was carried out before the experiment. The GO was used with a concentration of 100 ppm to account for the amount of GO in the actual beads. After 24 hours of dynamic contact at 150 rpm, the spent materials were filtered out, freeze-dried, and ground to obtain a fine powder, which was then analyzed by ATR-FTIR and XPS, as mentioned earlier. For the XPS analysis, a high-resolution scan was used. Statistical analyses of the curve fittings were done with the MATLAB software. Code is presented in the SI Text S1.

## Results and discussion

The detailed characterization of these beads using ATR-FTIR, scanning electron microscope (SEM), and digital images of GO, CS, CS-PEI, and CS-PEI-GO polymer beads are shown in the supporting information (Figure S1 and S2). The ATR-FTIR spectra of the GO, CS, CS-PEI, and CS-PEI-GO are shown in Figure S1, illustrating the functional variability of the beads and their successful synthesis. CS spectra showed main peaks at 1061 cm^−1^ for the C–O–C stretch, at 1656 cm^−1^ for the carbonyl stretch of the -NHCO- group resulting from the crosslinking^10^, and a weak broader peak in the range of 3100–3500 cm^−1^, which can be attributed to -OH and -NH_2_ groups^10,11^. In the CS-PEI material, the broad peak at 3100–3500 cm^−1^ was much stronger due to the inclusion of more -NH_2_ in the beads with the inclusion of PEI^3,10,11^. Furthermore, the same peak in the CS-PEI-GO spectrum was broader and stronger, due to the inclusion of GO. This can be easily understood with the peaks in the GO spectrum, where we can see a very strong broad peak in the range of 3000–3500 cm^−1^, corresponding to the hydroxyl stretching vibrations. Moreover, due to the crosslinking process in the CS-PEI-GO beads between the amine group from the CS and PEI, and –OH groups from GO, the peak at 3403 cm^−1^ decreases its intensity. Furthermore, the GO spectrum showed the characteristic peaks at 1044, 1156, 1622 and 1723 cm^−1^, which can be attributed to C-O stretching of alcohols, -C-O-C stretching of the ether groups, C = C bonds of the unoxidized hexagonal graphene structure, and the carboxylic groups, respectively^3,12–16^.

In the present study, after successful synthesis of the nanomaterials, they were exposed to 100 ppm Cr(VI) solution (pH = 3) to investigate the conversion of Cr (VI) to Cr(III). These conditions, *i.e*. pH and concentration of the chromium, were selected to have good signal/noise ratio for the XPS analysis and to have protonated amine and hydroxyl groups in the composite. These conditions were also previously described to be the best conditions for adsorption of Chromium^17–19^. Using a higher initial concentration of Cr(VI) (100 ppm) allowed us to achieve enough adsorption in the beads to be able to observe better peaks in the XPS spectra and characterize with higher accuracy the conversion of Cr(VI) to Cr(III)^7,20^.

The mechanism of Cr conversion was elucidated with the detailed use of XPS spectra and complemented by the ATR-FTIR spectroscopy. We also performed a detailed investigation of negative controls (CS, CS-PEI, and GO; note that PEI had to be combined with CS for analysis purposes since it is soluble in water) to determine the importance of functional variability in the successful uptake of Cr (VI) and reduction mechanisms of Cr (VI) to Cr (III).

**Figure 1:**
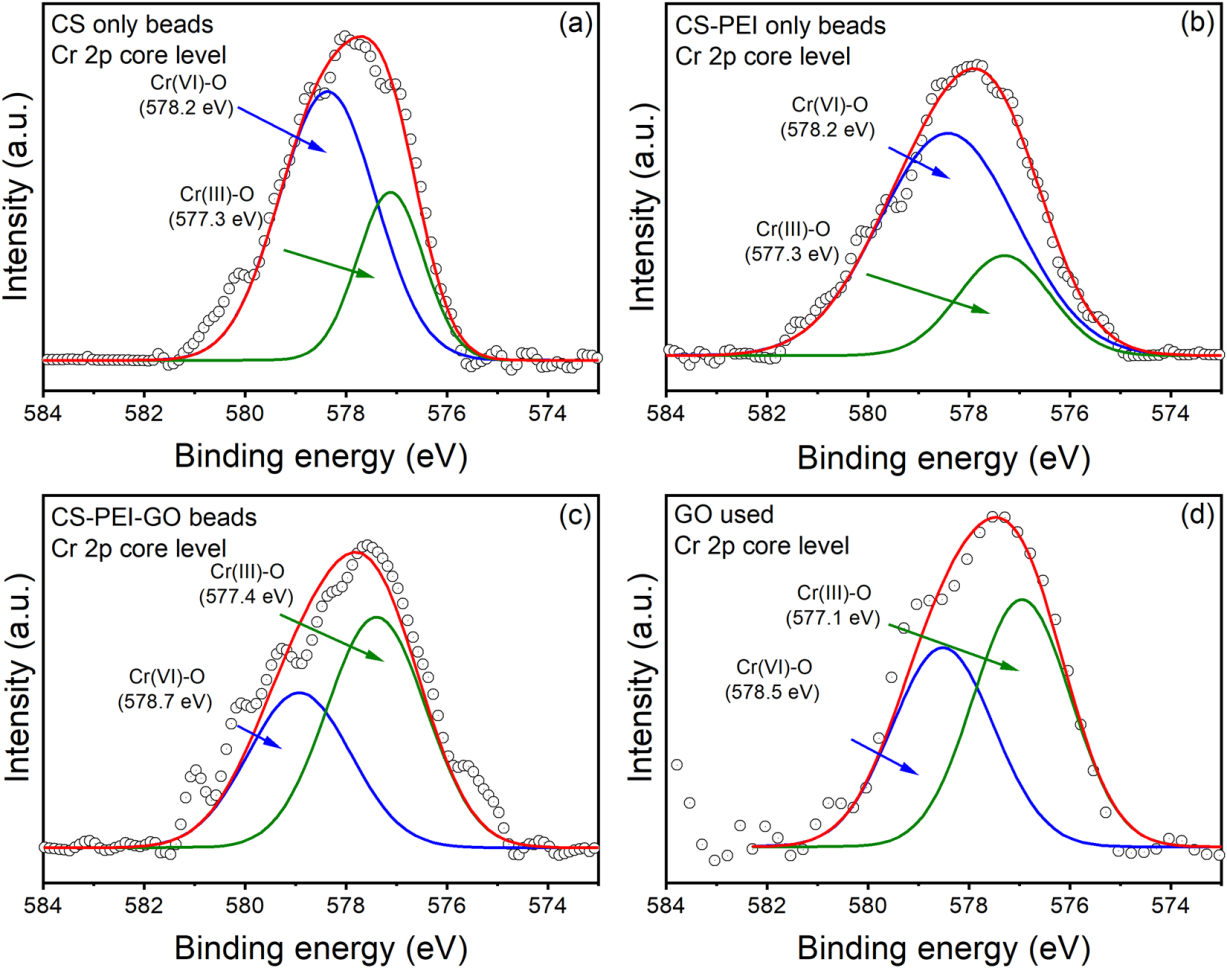
XPS spectra of Cr 2p3/2 for (**a**) CS, (**b**) CS-PEI, (**c**) CS-PEI-GO beads, and (**d**) GO after exposure to Cr (VI) solution. Experiments were performed at pH~3 with 100 ppm Cr (VI).

Figure 1 shows the high-resolution XPS spectra of Cr2p for all four powdered materials. The deconvolution results of the Cr2p3/2 peak, which is composed of two peaks at 577 and 578, were assigned to Cr (III) and Cr (VI), respectively^5,21^. The presence of Cr (VI) and Cr (III) based on the areas of the XPS curve fittings are shown in the Table 1.

**Table 1:** Percentage abundance of Cr (VI) and Cr (III) based on the area of the fitted XPS peaks.

Cr Species	CS	GO	CS-PEI	CS-PEI-GO
Cr (VI)	70%	46%	77%	41%
Cr (III)	30%	54%	23%	59%

In the results, CS-PEI-GO beads presented a more pronounced production of Cr (III) based on the intensity of the XPS peak. Based on Table 1, the type of materials influenced the abundance of Cr (III) produced. The reduction of Cr (III) increased with the following material order investigated CS-PEI < CS < GO < CS-PEI-GO. The inclusion of GO resulted in more dominant signals of Cr (III), as indicated by Fig. 1d. Based on these results, the trends in Cr speciation with each functional group of the polymer beads were further investigated via XPS and ATR-FTIR. Using further detailed XPS analysis of the changes in the functional groups of the different materials exposed to Cr (VI) and by determining their potential chemical reaction equations, we were able to determine the mechanism of conversion of Cr (VI) to Cr (III) as explained below:

Chemical species of Cr (VI) depends on pH and their concentrations, as shown in the speciation diagram of chromium (Figure S3). The different chemical species range from CrO_4_^2−^ at pH above 6 through HCrO_4_^−^ and Cr_2_O_7_^2−^ at pH below 6.5 to H_2_CrO_4_ at pH <0.7.

It is known that when dichromate (Cr_2_O_7_^2−^) ions are dissolved in water, they ionize to form chromate (CrO_4_^2−^) ions and H^+^ as given by Eq. (1)^22,23^.

1 $${\text{Cr}}_{2}{\text{O}}_{7}^{2-}+{\text{H}}_{2}\text{O}\rightleftarrows 2{\text{CrO}}_{4}^{2-}+2{\text{H}}^{+}$$

which is a reversible reaction and highly dependent on pH and analytical concentration of chromium. At the pH studied here, pH 3, the predominant species are HCrO_4_^−^ and ${\text{Cr}}_{2}{\text{O}}_{7}^{2-}$.

In solutions, CrO_4_^2−^ form an equilibrium with HCrO_4_^−^, which is the most dominant Cr (VI) species when pH of the solution is between 3 and 6, which was the pH used in this study^24,25^. The equilibrium equation is given as:

2 $${\text{CrO}}_{4}^{2-}+{\text{H}}^{+}\rightleftarrows {\text{HCrO}}_{4}^{-}$$

The Cr (VI) species in solution are expected to interact with CS through amines (-NH_2_), hydroxyl (-OH) and primary alcoholic groups (-CH_2_OH)^3^.

In the currently investigated pH, the amines in the CS will follow the following equation at acidic pH:

3 $$\text{R}-{\text{NH}}_{2}+{\text{H}}^{+}\to \text{R}-{\text{NH}}_{3}^{+}$$

which suggests that the -NH_2_ groups in CS will also remain positively charged^24,25^. Therefore, it can be theorized that hydroxyl and amine protonated groups in CS are responsible for the removal of Cr (VI) species from the aqueous solution by forming electrostatic attractions with the negatively charged Cr(VI) species^26,27^ thereby, removing them from the solution, resulting in Cr (VI) signals as presented in Fig. 1. Therefore, ${\text{HCrO}}_{4}^{-}$ would bind to positively charged functional groups on the beads according to the equations below:

4 $$\text{R}-\text{OH}+{\text{HCrO}}_{4}^{-}\to \text{R}-\text{OH}\cdot \cdot \cdot \cdot {\text{HCrO}}_{4}^{-}$$

5 $$\text{R}-{\text{NH}}_{3}^{+}+{\text{HCrO}}_{4}^{-}\to \text{R}-{\text{NH}}_{3}^{+}\cdot \cdot \cdot \cdot {\text{HCrO}}_{4}^{-}$$

By analyzing the ζ-potential of the materials, it was possible to provide data on the surface charge of the polymer beads. For the unused beads in DI water, we can see positive surface charge density, as shown in Table S1. Positive zeta potentials indicate favorable conditions for the formation of electrostatic attractions with anionic species, such as CrO_4_^2−^ (Cr(VI)). After adsorption, the surface charge was still positive but became less positive after interaction with the negatively charged Cr(VI) species. Positive values are an indicative of the protonation of the functional groups on the surface of the polymer beads that could lead to electrostatic interactions.

In the XPS (Fig. 1), the Cr (VI) signals observed are linked to the interaction of ${\text{HCrO}}_{4}^{-}$ with the amines. This interaction does not change the oxidation state of Cr species.

As suggested by the redox potentials of ${\text{HCrO}}_{4}^{-}$ (+1.35) and CrO_4_^2−^ (−0.13), ${\text{HCrO}}_{4}^{-}$ is highly reducible. Therefore, in the presence of an electron donor, the reduction reaction can occur according to the following equation,

6 $${\text{HCrO}}_{4}^{-}+7{\text{H}}^{+}+3{\text{e}}^{-}\rightleftarrows {\text{Cr}}^{3+}+4{\text{H}}_{2}\text{O}$$

resulting in the conversion of Cr (VI) to Cr (III), which is a less toxic form of chromium^24^. Based on the chemical groups available on CS beads and the changes in the abundance of functional groups (Table 1), the following redox reactions are suggested to be involved in the reduction of Cr(VI) to Cr(III)^24,28–30^.

7 $$\text{R}-{\text{CH}}_{2}-\text{OH}+\text{Cr}(\text{VI})\to \text{R}-(\text{C}=\text{O})\text{H}+\text{Cr}(\text{III})$$

8 $$\text{R}-(\text{C}=\text{O})\text{H}+\text{Cr}(\text{VI})\to \text{R}-\text{COOH}+\text{Cr}(\text{III})$$

In addition to the oxidative reactions described above, it is possible that there can be oxidation of -CH_3_ groups causing Cr (VI) reduction, which can be represented by the following equation^24,28^.

9 $$\text{R}-{\text{CH}}_{2}-\text{H}+\text{Cr}(\text{VI})\to \text{R}-{\text{CH}}_{2}-\text{OH}+\text{Cr}(\text{III})$$

The formed hydroxyl group, according to Eq. (9), can be further oxidized as given by Eqs. (7–9)^24,28^.

**Figure 2:**
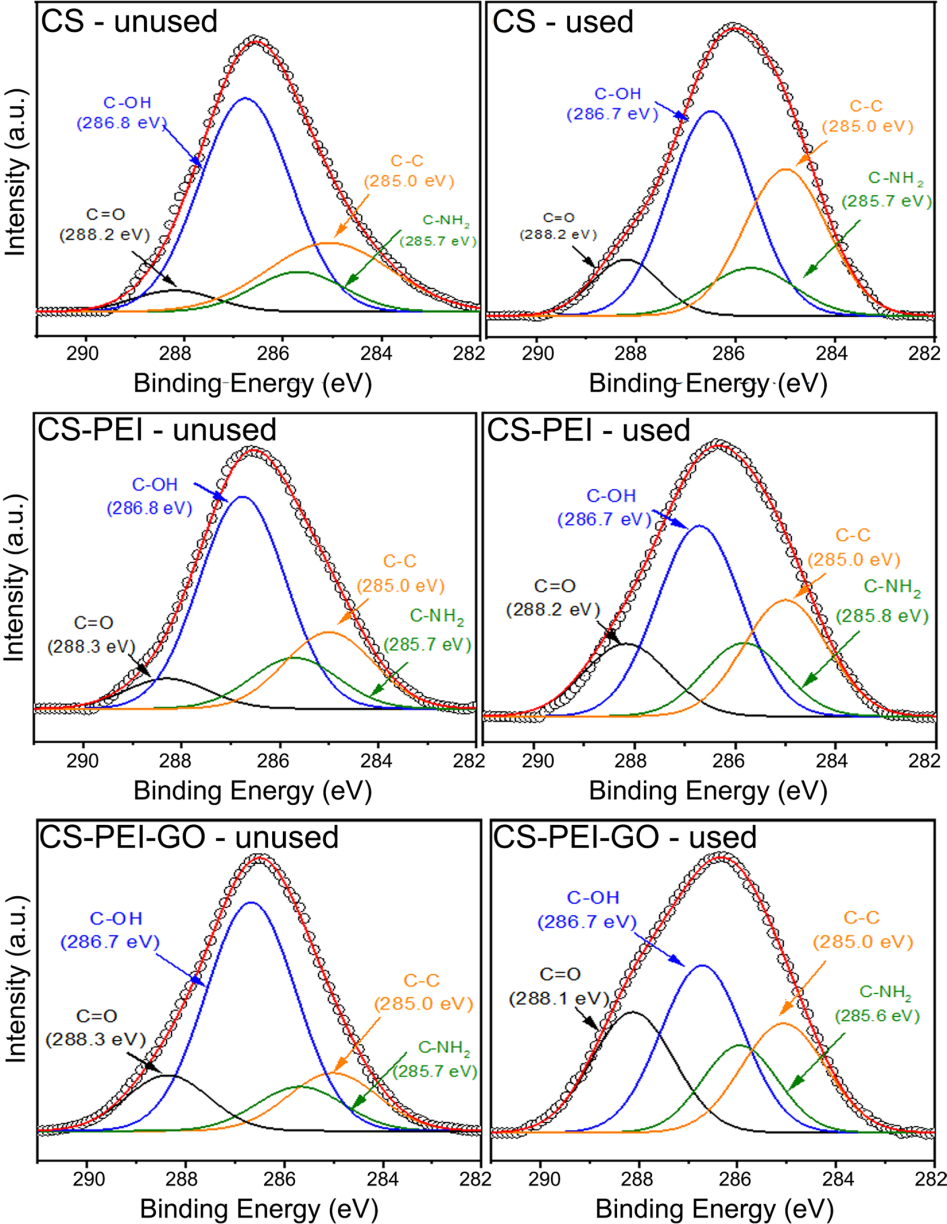
XPS spectra showing deconvolution of C1s core level for unused and used polymer beads. Experiments were performed at pH~3 with 100 ppm Cr (VI).

The quantitative analysis of the XPS spectra of the unused and used CS showed more evidence of these chemical reactions (Eqs. 7–9). As shown in Fig. 2, the C1s peak can be deconvoluted into four main peaks at 285.0, 285.7, 286.7 or 286.8, and 288.2 eV^31–33^. The peak at 285.0 can be assigned to the C-C bond from chitosan, and the peak at 285.7 eV can be assigned to the C-NH_2_ bond, which is generated from the amine groups in CS. The later does not seem to be affected by the Cr adsorption. Table 2 shows the % of the different functional groups involved in the redox mechanisms. As it is observed for all the samples, a conversion from –OH to carbonyl group is happening. Furthermore, this increase is more prominent in the CS-PEI-GO, due to higher amounts of hydroxyl functional groups coming from the addition of GO to the polymer beads. In the spectrum of the unused beads (Fig. 2), the peak at 286.7 eV is more pronounced than the other peaks (59%) and can be attributed to the C-OH bond, which is generated due to the presence of hydroxyl bond and epoxy bonds in CS. While the intensity of the -OH peak in CS reduced to 46%, the peak attributing to the C=O, which is at 288.2 eV, increased from 5% to 10% indicating the occurrence of oxidation reactions suggested in Eqs. (7) and (8)^24^.

**Table 2:** Percentage abundance of different bonds based on the area of the fitted XPS peaks for the unused and used polymer beads.

Sample	Condition	C-OH	C=O	C-C/ C=C	C-NH_2_
CS	Unused	59%	5%	25%	11%
	Used	46%	10%	33%	11%
CS-PEI	Unused	57%	8%	21%	14%
	Used	42%	16%	26%	16%
CS-PEI-GO	Unused	61%	13%	15%	11%
	Used	35%	25%	23%	17%
GO	Unused*	52%	22%	21%	—
	Used	37%	28%	35%	—

*For the unused GO, the contribution of the π-π shakeup satellite is estimated 5%.

When considering CS-PEI beads, the addition of PEI provides further -NH_2_ groups on the bead surface, as suggested by the peak at 285.7 eV, which was more pronounced compared to the CS. The inclusion of PEI accounts for the -NH_2_ groups that are spent during the crosslinking process of CS with GLA. The other peaks at 285.0, 286.7 or 286.8 and 288.2 or 288.3 eV can be attributed to C-C, C-OH and C=O bonds from CS, respectively, as previously explained^31–33^. Compared to unused CS, unused CS-PEI beads show a more pronounced peak for -C=O due to the crosslinking with GLA. However, following the adsorption of Cr (VI), the -C=O peak becomes even more pronounced, indicating a similar reduction to what was observed with CS beads described earlier. It is also important to note that, compared to CS, the CS-PEI beads showed a less pronounced Cr(III)-O peak, as shown in Figs. 1a,1b. This decreased reduction from Cr (VI) to Cr (III) can be attributed to the limited amount of -OH groups (or electron donors) lost during the crosslinking of CS with PEI in the presence of GLA. Hence, this limited amount of -OH groups in the CS-PEI led to a declined reduction of Cr (VI) to Cr (III). Ultimately, this led to a higher Cr (VI) signal, as shown in Table 1. This observation also agrees with the more significant peak shown for C-NH_2_, indicating the contribution of this functional group to the Cr (VI) uptake based on the Eq. (5), but lack of reduction ability by this functional group^3,34,35^.

Once GO was included in the CS-PEI polymer, a higher contribution of Cr (III) was observed compared to the other polymer beads. GO possesses more oxygen-containing functional groups. In addition to -OH and -CH_3_ functional groups, GO also contains -COOH and -C=O groups that can be protonated when present in an acidic environment. Additionally, the inclusion of GO in the CS-PEI beads provides more oxygen functional groups that can be oxidized. Therefore, more Cr (VI) will go through redox reactions as suggested by Eqs. (7–9). This can be seen by the more pronounced Cr (III) signals in the CS-PEI-GO as shown in Table 1, which clearly indicates the importance of the inclusion of GO in this redox process.

These potential mechanisms are well described by the C1s core level XPS spectra of used CS-PEI-GO beads as shown in Fig. 2 and Table 2. Compared to CS and CS-PEI, the peak at 288.3 eV, which was attributed to C=O, became more pronounced after the inclusion of GO^4,36,37^. The intensity of -C=O peak increased further after the adsorption, indicating oxidation of R-OH and -CH_3_, as suggested by Eqs. (7–9). The hydroxyl group can be oxidized into an aldehyde or carboxylic acid while the Cr (VI) is reduced to Cr (III). For clarification, for the unused GO, the contribution is not 100% because in Table 2 is not showing the % of the π-π shake up satellite. After adsorption, the results showed a positive correlation between the increase in the XPS Cr (III) signal and –C=O signals in the ATR-FTIR, while Cr (VI) signal decreased, indicating conversion of Cr(VI) to Cr(III) by the redox reaction of -OH groups

Furthermore, the oxidation of -OH groups can be justified with the observation of a smaller peak for -C-OH at 286.7 eV as shown in Fig. 2 and Table 2 compared to the control beads. As presented in Table 1 and Eqs. (7–9), the reduction of Cr (VI) to Cr (III) species were justified by the more pronounced Cr(III)-O peak at 577.4 eV (Fig. 1).

**Figure 3:**
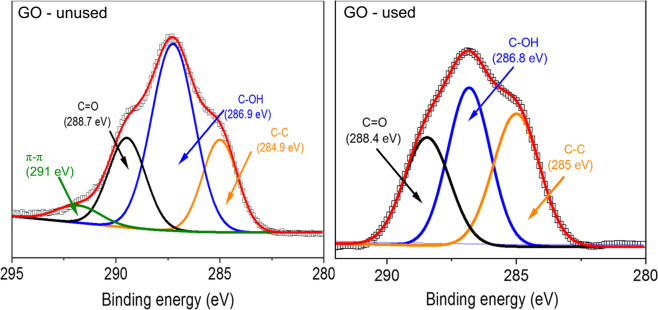
XPS spectra showing deconvolution of C1s core level for unused and used GO. Experiments were performed at pH~3 with 100 ppm Cr(VI).

Therefore, it is clear that GO contains more potential to uptake Cr (VI), followed by its conversion to Cr(III). Thus, CS-PEI-GO beads are more efficient in not only removing Cr (VI) but also in converting it to a less toxic form of chromium (Cr(III)) when compared to the control beads^3,34^. This is further confirmed by the XPS spectra of unused and used GO showing loss of -OH groups as shown in Fig. 3.

Similar to the results obtained by XPS, the qualitative analysis of the ATR-FTIR of the used beads showed evidence of the generation of new carboxylic groups as observed in Figure S4. Figure S5 showed appreciable changes in the ATR-FTIR spectra for the unused and used CS-PEI-GO. For comparison, spectra were normalized to the C–O–C stretch (1061 cm^−1^). In the used CS-PEI-GO composite, the carbonyl stretching peak at 1651 cm^−1^ showed an increased intensity. This increase in the intensity suggested oxidation of the functional groups, which was also confirmed by XPS (Fig. 2), and explained by the reactions in Eqs. (7) and (8). Furthermore, the peak of the C-H stretching at 2848 cm^−1^ became less visible in the used CS-PEI-GO due to the oxidation of the C-H of the chitosan (Eq. 9). When the C-H functional group was oxidized, the hydroxyl band observed in the ATR-FTIR (Figure S4) spectra became more intense.

## Conclusions

This research reports a comprehensive breakdown of the possible mechanisms of Cr uptake and speciation. The results showed that CS-PEI-GO polymer beads possessed more potential to uptake Cr (VI) species and convert them to a less toxic Cr (III) species compared to the CS and CS-PEI beads. In summary, functional groups such as -OH and -NH_2_ found in CS and CS-PEI beads, under acidic conditions, will get protonated and bind to Cr (VI) species. After this, -OH groups will further act as electron donors to facilitate the reduction of Cr (VI) to Cr (III). We proved that –OH functional groups in the composite play a major role in the conversion mechanisms of Cr (VI) to Cr (III).

The addition of GO to the polymer composite (CS-PEI) can increase the uptake of Cr (VI) ions, as protonation of additional -OH, -(C=O)H, and -COOH groups in GO will result in enhanced electrostatic attractions. Furthermore, GO showed a better reduction of Cr (VI) as suggested by the loss of -OH groups and production of –(C=O)H and –C-H due to the oxidation of -OH groups (Eqs. 7–9).

The process of reducing Cr (VI) to Cr (III) is beneficial in the sense that Cr (III) is a less toxic species. This is important when it comes to not only remove Cr (VI) from aqueous solutions but also in disposing of spent adsorbent materials or regenerating the adsorption sites by a chemical reagent for extended use of beads due to the reduced toxicity levels of the Cr contaminants. In such sense, the inclusion of GO in the polymeric matrix provides insight into a more practical and sustainable water treatment processes that involve treating Cr contaminated water.

## Associated Content

### Supporting Information

Electronic Supplementary Information (ESI) available: Figure S1 and S2 showed the ATR-FTIR, SEM, and digital images for all the polymer beads prepared, Figure S3 showed the speciation diagram for chromium, Table S1 showed the ζ-potential measurements, Figures S4 and S5 showed the ATR-FTIR spectra of the used polymer beads, and Text S1 showed the complementary MATLAB analysis of the XPS curves.

## Supplementary information

Supplementary Information.
