# Supplementary material for: Redox mechanisms of conversion of Cr(VI) to Cr(III) by graphene oxide-polymer composite
Source: Sci Rep. 2020 Jun 8;10:9237. doi: 10.1038/s41598-020-65534-8 (PMC7280210; doi:10.1038/s41598-020-65534-8)
Supplement: Supplementary file 1 — Supplementary Information. [file 41598_2020_65534_MOESM1_ESM.docx]

**Supporting information**

Redox mechanisms of conversion of Cr(VI) to Cr(III) by graphene oxide-polymer composite

Pasan Chinthana Bandara,^a,Φ^ Janire Peña-Bahamonde, ^a,Φ^ and Debora Frigi Rodrigues ^*a^

^a^ Department of Civil and Environmental Engineering, University of Houston, Houston, TX 77204-4003.

Email: [dfrigirodrigues@uh.edu](mailto:dfrigirodrigues@uh.edu)

Figure S1. ATR-FTIR spectra of CS, CS-PEI, and CS-PEI-GO beads, and GO showing the important peaks indicating their functional variability.


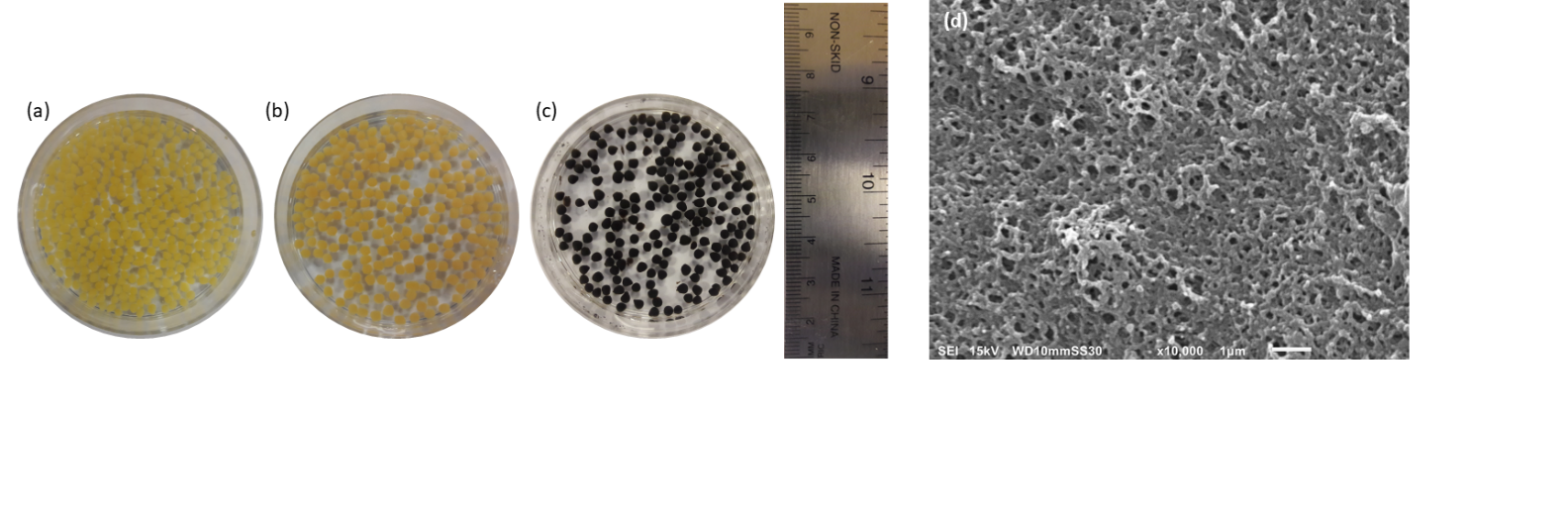


**Figure S2.** Digital photos of (a) CS, (a) CS-PEI, and (c) CS-PEI-GO beads and SEM image of the surface of the CS-PEI-GO beads.

**
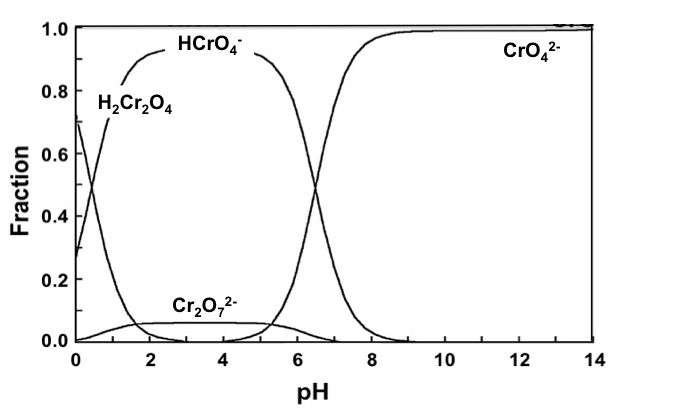
**

**Figure S3:** Speciation diagram of chromium (VI). Adapted from (1).

**Table S1:** ζ-potential measurements before and after exposition with Cr (VI).

| **Sample** | ζ**-potential** | |
| --- | --- | --- |
|  | **Before exposition** | **After exposition Cr(VI)** |
| CS | 42.4 ± 0.6 | 18.0 ± 1.1 |
| CS-PEI | 32.0 ± 1.9 | 14.0 ± 0.4 |
| CS-PEI-GO | 41.2 ± 0.9 | 24.0 ± 0.8 |

**Text S1:** Complementary XPS curve fitting with MATLAB.

To complement the XPS curve fitting by MultiPak V7.0.1 (ULVAC-PHI, Inc.) and Origin Pro8.5 (OriginLab, Northampton, MA), alternative curve fittings were done with the MATLAB using the code given below.

clear all;

datavec = csvread('C:\CS.csv');

gmInitialVariance = 0.1;

Mu = [288.2;286.8;285.7;285.0];

Sigma = cat(3,gmInitialVariance,gmInitialVariance,gmInitialVariance,gmInitialVariance);

initialWeights = [0.25 0.25 0.25 0.25];

S = struct('mu',Mu,'Sigma',Sigma,'ComponentProportion',initialWeights);

output = zeros(100,4);

for i = 1:100

    f = fitgmdist(datavec,4,'Options',statset('MaxIter',i),'Start',S);

    output(i,1)=[f.mu](http://f.mu/)(1);

    output(i,2)=[f.mu](http://f.mu/)(2);

    output(i,3)=[f.mu](http://f.mu/)(3);

    output(i,4)=[f.mu](http://f.mu/)(4);

end

Smoothen experimental data from the MultiPak for each sample was used as the raw data for each curve fitting, which was converted to a single column data set using R-studio. The MATLAB inbuilt function fitgmdist was used to create a mixture design of gaussian distributions to fit the experimental data. The mean values from the XPS curve fitting from MultiPak and Origin were used as the initial conditions for each peak in MATLAB function, in order to find the deviation of the position of the peak of interest. The number of iterations was fixed at 100, which was adequate to reach the optimum solution for all the considered cases.

By comparing the mean values from the MATLAB function vs the mean values from MultiPak and Origin, it was found out that the mean values differ only by a margin of ±0.2% indicating the accuracy of the XPS curve fittings as suggested by the lower χ^2^ values and Adjusted R^2^ values which are close to unity.

Figure S4. ATR-FTIR spectra of the CS, CS-PEI, and CS-PEI-GO beads after the adsorption of Cr.

Figure S5. ATR-FTIR spectra of the unused and used CS-PEI-GO. The arrow indicated the C-H stretching.

**Reference:**

(1) Gherasim, C.-V.; Bourceanu, G.; Olariu, R.-I.; Arsene, C. A Novel Polymer Inclusion Membrane Applied in Chromium (VI) Separation from Aqueous Solutions. *J. Hazard. Mater.* **2011**, *197*, 244–253.
